# Enabling six- to ten-year-old children to self-report their wellbeing and quality of life: development and psychometric investigation of an age-adapted and video-assisted version of the KIDSCREEN-27

**DOI:** 10.1007/s11136-025-03939-6

**Published:** 2025-03-01

**Authors:** Mette Kurtzhals, Paulina Sander Melby, Peter Elsborg, Peter Bentsen, Caroline Eckert, Malte Nejst Larsen, Glen Nielsen

**Affiliations:** 1 Department of Nutrition, Exercise and Sports, University of Copenhagen, Copenhagen N, Denmark; 2 Center for Clinical Research and Prevention, Copenhagen University Hospital – Bispebjerg and Frederiksberg, Nordre Fasanvej 57, Frederiksberg, 2000 Denmark; 3 Department of Sports Science and Clinical Biomechanics, University of Southern Denmark, Odense, Denmark; 4 Department of Geoscience and Natural Resource Management, University of Copenhagen, Frederiksberg, Denmark

**Keywords:** Validation, Measurement, Mental health, Preschool, Schoolchildren

## Abstract

**Purpose:**

Identifying the underlying factors that contribute to poor wellbeing and developing strategies for early intervention are essential for promoting overall wellbeing. Many important aspects of wellbeing and quality of life are subjective experiences and therefore require self-report. The KIDSCREEN-27 questionnaire is widely used for this purpose. However, the self-report versions have mainly been validated for children aged 12 to 18 years. This study aims to develop a video-assisted format the KIDSCREEN-27 that enable self-report of wellbeing by children aged six to ten years and to test its psychometric properties.

**Methods:**

The Danish-translated version KIDSCREEN-27 was slightly adapted in wording and items (N=12) and a video-format, including audio, illustrations, and smiley-supported scales, was developed, and tested. Next, a psychometric investigation of this version (KIDSCREEN-VIDEO) was conducted on 788 Danish children aged six to ten years (49.8% girls).

**Results:**

Confirmatory factor analysis showed an acceptable to good model-fit: *X*^2^ = 727.053; df = 242; P <0.001; root mean squared error of approximation=0.05; the comparative fit index = 0.98; and the Tucker-Lewis index = 0.98, and factor loadings ranged from 0.40 to 0.88. Cronbach’s alpha values ranged from 0.65 to 0.89, suggesting acceptable to good internal reliability of the scales. Linear mixed model analyses, and Pearson’s r correlation coefficients showed positive associations with the global and physical self-worth scales, indicating convergent validity. The test for measurement invariance indicated the model fit for the five-factor model was consistent across sex and age groups.

**Conclusion:**

Based on our results, the KIDSCREEN-VIDEO provides a promising self-reported measure for wellbeing among children aged six to ten.

**Supplementary Information:**

The online version contains supplementary material available at 10.1007/s11136-025-03939-6.

## Background

Wellbeing and health-related quality of life (HRQoL) are important parts of positive child development and health [1]. Poor wellbeing among children and youth is an increasing concern worldwide [2], and in Denmark, a decrease in wellbeing has also been noted in recent years [3–5]. It is important to identify the underlying factors contributing to poor wellbeing in order to develop strategies for early prevention and the promotion of wellbeing and HRQoL. For this purpose, wellbeing and HRQoL measures with strong psychometric properties are crucial for monitoring and evaluating the effects of interventions aiming to improve children’s wellbeing.

Children’s wellbeing has been studied across a wide range of disciplines, cultures, communities, and countries, resulting in an assortment of definitions and measurements [6, 7]. In public health research, wellbeing has been defined and measured as consisting of different but sometimes overlapping aspects, such as an inherently positive state (e.g., happiness) or absence of poor mental health (e.g., low levels of stress) or psychological aspects of these constructs (e.g., self-esteem) [6]. Other conceptualizations and definitions are more based on wellbeing as functioning well (e.g., absence of behavioral difficulties) in everyday life [8]. Despite the lack of a single consistent definition, the academic literature within health promotion often defines wellbeing in a way that echoes the World Health Organization’s (WHO) [6, 9] definition of health as “*a state of complete physical*,* mental*,* and social wellbeing and not merely the absence of disease or infirmity*”. The concept of quality of life is also closely associated with this definition. Yet, beyond the feelings of physical, mental, and social wellbeing, the concept of quality of life encompasses more concrete aspects that are important for overall wellbeing in everyday life settings (e.g., physical surroundings) [10]. This variation in definitions is mirrored in a range of measurements evaluating different dimensions of wellbeing, mental health, and/or quality of life [7].

A seminal research endeavor that comprehensively investigated aspects of children’s wellbeing and HRQoL across 13 European countries is the KIDSCREEN project. The project developed three measures of wellbeing and HRQoL for children and adolescents aged eight to 18 years, intended for use in epidemiological population health surveys, intervention studies, and research projects with cross-cultural applicability [11]. Conceptually, the KIDSCREEN instruments are based on a definition of HRQoL as a multidimensional construct consisting of physical, emotional, mental, social, and behavioral components of wellbeing in different everyday contexts [11], thus, it effectively encompasses both wellbeing and HRQoL.

The KIDSCREEN instruments include both self-report and proxy-report measures of different lengths, each with a one-week recall period [12–15]. The longest version, KIDSCREEN-52, measures ten dimensions of wellbeing and HRQoL: Physical Wellbeing, Psychological Wellbeing, Moods and Emotions, Self-Perception, Autonomy, Parent Relations and Home Life, Financial Resources, Peers and Social Support, School Environment, and Bullying [12]. KIDSCREEN-27 was developed as a condensed version, with minimal information loss while maintaining strong psychometric properties and measuring five dimensions of wellbeing and HRQoL. KIDSCREEN-10 is a further condensed version that measures a single unidimensional index and provides a global score of HRQoL based on ten items from the five dimensions of the longer version [12]. In studies that include several psychometric measures (as is often the case) keeping a questionnaire as short as possible is important for ensuring concentration and retention, especially when surveying children. However, given the complexity and multifaceted nature of wellbeing, the ability to measure different aspects is also crucial [6, 7]. For these reasons, we find that KIDSCREEN-27 often will be a suitable option for research projects focusing on children’s wellbeing.

KIDSCREEN-27 measures the following five dimensions of wellbeing and HRQoL: Physical Wellbeing (5 items), Psychological Wellbeing (7 items), Autonomy and Parent Relation (7 items), Peers and Social Support (4 items), and School Environment (4 items). The Physical Wellbeing dimension measures the level of the individual’s physical activity and energy as well as the extent to which a child feels well and perceives their general health. Psychological Wellbeing assesses the level of positive emotions and satisfaction with one’s life as well as the absence of negative feelings such as loneliness and sadness. Autonomy and Parent Relation involves the quality of the interaction between child and parent/caregiver as well as whether the individual feels loved and supported by the family. It also assesses the individual’s perceived level of autonomy and the perceived quality of the financial resources of the child. Peers and Social Support measures the extent and quality of the interaction between the child and peers/friends as well as their perceived peer/friend support. The School Environment dimension assesses the individual’s perception of his/her attention in a class setting and his/her feelings of happiness during the school day as well as the perception of getting along with their teachers [11].

Self-reported versions of KIDSCREEN have mainly been validated and used in ages 12 to 18 years [16–21]. A 2023 review showed that a greater number of studies assess wellbeing and HRQoL among healthy children aged 12 to 18 years using a self-report version alone, compared to children aged six to 11 years (i.e., 277 studies vs. 170 studies) [20]. Moreover, only 12 of 226 studies were found to include KIDSCREEN self-reporting alone for children aged six and seven [20]. Studies that rely solely on KIDSCREEN self-reporting for this young age group may face scrutiny, as the self-report versions of the KIDSCREEN instruments are currently only validated for children older than 11 years. Additionally, children with reading challenges are likely to require help from an adult when answering.

For these reasons, parent or teacher proxy-reporting is often used for children under the age of eight [22], meaning that the wellbeing measures for children this age are based on adults’ perspectives and interpretations of various dimensions of the child’s wellbeing [22, 23]. However, it can be argued that children are the most qualified experts, or at least important informants, regarding their own feelings of physical and psychological wellbeing as well as how they feel in their relations with friends, parents, and in the school. Studies show differences between how children and their parents perceive and report the child’s wellbeing [22, 24], a finding that also has been identified with the KIDSCREEN instruments [18, 25, 26]. Thus, to assess wellbeing among six- to ten-year-old children from their own perspective, there is a need for a self-report measure with robust psychometric properties, which might necessitate more age-appropriate format and wording.

Some studies have utilized the KIDSCREEN self-reporting for children aged six to seven; however, these studies did not conduct an evaluation of the psychometric properties for this age-group [20]. Research suggests that children older than seven years, potentially as young as age five, can provide reliable self-reports of their health status, when a questionnaire is age-appropriate [23]. Yet, children at different ages may understand and interpret questions differently [27]. This is particularly relevant given the wide age range of respondents for the KIDSCREEN instruments, which span from eight to 18 years, as it may present challenges, especially for younger children and those with reading difficulties. To our knowledge, the psychometric properties of a self-report version of KIDSCREEN-27 for children younger than eight years have only been investigated in a sample of 256 Romanian children aged six years [28]. However, this study did not evaluate the factor structure, but only used Pearson correlations, Cronbach alpha values, and interclass correlations as indicators of the psychometric properties. Furthermore, the results showed low Cronbach’s alpha values of 0.55 to 0.66 indicating that some items did not work well for this age group [28]. Self-report measures for young children, need to consider the cognitive development, reading abilities, and emotional maturity, as well as to provide concrete support for the children’s answering process, when using, developing, or adapting an instrument to enable them to self-report on their wellbeing [29, 30].

In Denmark, children begin school at age six and only a few can read well at this age. Even as late as 4th grade (age ten), about 20% of the Danish children have challenges with reading, which is similar to many other countries [31]. To enable Danish children aged six to ten – including those with limited reading abilities – to report their own wellbeing and HRQoL, this study purposes to test a video-assisted and age-adjusted version of the Danish, non-validated KIDSCREEN-27 questionnaire. To our knowledge, no video-formatted KIDSCREEN instrument has yet been investigated or developed, nor have we identified any studies evaluating the psychometric properties of the KIDSCREEN-27 for Danish children. Thus, the aims of the study are twofold: (1) to develop a video-assisted format of the KIDSCREEN-27 questionnaire that enable self-reporting by Danish children aged six to ten years, and (2) to investigate the psychometric properties of this questionnaire among Danish children aged six to ten years.

## Methods

### Procedure

The adaptation process was based on the steps and guidelines outlined by WHO: ‘Process for translation and adaptation of instruments’ [32]. The first phase was to age-adapt the existing Danish translated version of KIDSCREEN-27 [33] by changing single words, phrases, or whole sentences, when necessary for the children’s comprehension, development, and emotional maturity. Next, an adaption of the existing pencil-paper format to an electronic format, was undertaken to support child self-report for ages six to ten. The second phase was to pilot-test and conduct cognitive interviews with age-appropriate children, to gain a better understanding of their experiences and perceptions of the adaptions of both format and items, resulting in a discussion of the appropriateness of the preliminary adjustments. The final phase was to test the age-adapted and video-assisted version and investigate the psychometric properties.

All participants completed the questionnaires on tablets while wearing headphones at their desks in the classroom. Beforehand, a brief oral introduction was given to the children by a researcher, informing the children that the questionnaire was not a test, meaning there were no right or wrong answers. This was followed by a short video-assisted introduction, illustrating how to play the videos, answer a question, and continue to the next question. In order to ensure there was no missing data on any items, children were required to answer each question before proceeding to the next question. During data collection, sufficient time was provided to enable all children to complete the survey. The children were instructed to raise their hands if they needed help understanding a question, the response options, or proceeding to the next question. Thus, when needed, a researcher would actively assist the children with either comprehension or technical issues. In the KIDSCREEN-27 questionnaire, items are answered on a 5-point response scale with higher scores indicating a better wellbeing and HRQoL [11].

### Phase 1: initial age- and format-adaptation

Two researchers (MK and PM) with Danish as their first language, independently revised and age-adjusted the current and original Danish-translated version of KIDSCREEN-27 [33], which has not been validated in a Danish context. The focus was to consider whether the items’ wordings and answer scale format could be made more age-appropriate for children aged six to ten years, without changing the meaning or factor indication of the items. Next, the age-adjusted versions were compared and combined into a single version, through a joint discussion between the two researchers and a third researcher (GN) with expertise in wellbeing, and psychometrics. To ensure age-appropriateness, the project group then tested and discussed the questionnaire with a small convenience sample of children within the age group. Informed consent was obtained from all legal guardians before the discussion with their children. In these discussions, items that the children misunderstood or found difficult to understand were discovered; improvements were then identified by discussing these with the children. Based on these steps, the project group made six minor adjustments to the original Danish version. This included in items 10, 12, 23, 25; as well, 26 single words were changed or added. For example, in item 10, “Have you felt so bad that you didn’t feel like doing anything?”, the word ‘bad’ was translated to ‘dårligt’ in the original Danish version of KIDSCREEN-27. However, the children interpreted ‘dårligt’ (*‘bad’*) as referring to being physically ill. Therefore, we replaced the word ‘dårligt’ with ‘ked af det’, which also refers to the sense of feeling bad but in a way that more specifically relates to mood and emotions (i.e., *‘feeling low’* or *‘feeling down’*). The largest change was for item 1, where we found it necessary to rephrase the item from “In general, how would you say your health is (‘*Hvordan er din sundhed generelt*’)?” to “How are you feeling/doing (‘*Hvordan har du det*’)?”, in order to better enable the young age group to understand the question. A complete list of specific adjustments can be found in Appendix 4.

Based on this, a preliminary video-assisted format of the questionnaire in terms of structure, illustrations, and “smiley-face” supported scales was developed. To our knowledge, no guidelines, or frameworks for technology adaptations of video-assisted questionnaires exist. However, research has shown that electronic and paper-pencil questionnaire formats display equivalent scores across age groups and platforms e.g., computers and tablets [34–36] and indicates that utilizing an electronic format supports self-reporting among children [23]. Bevans et al. [37] provide a framework for selecting child-report health assessment instruments and describe different assessment strategies that capitalize on children’s cognitive strengths while accommodating for their cognitive limitations at different developmental stages. The development process of the video-assisted format was guided by the strategy for children aged six to 11 years, as outlined by Bevans et al.: “Assessment tools must be tested to ensure that items are understandable to children. Illustrated items or response categories may improve interest, attention, and engagement in the assessment process”. Items should be read aloud to children or administered via ACASI [Audio-Computer Assisted Self- Interview] format to compensate for diversity in reading capabilities” [37] (pp. 14). Adequate time needed for completion must be allocated [37]. The development of the video-assisted format was also inspired by Elsborg et al. [38, 39], who successfully used video-assisted self-reported questionnaires for measuring physical literacy among Danish children aged seven to 12 years.

**Fig. 1:**
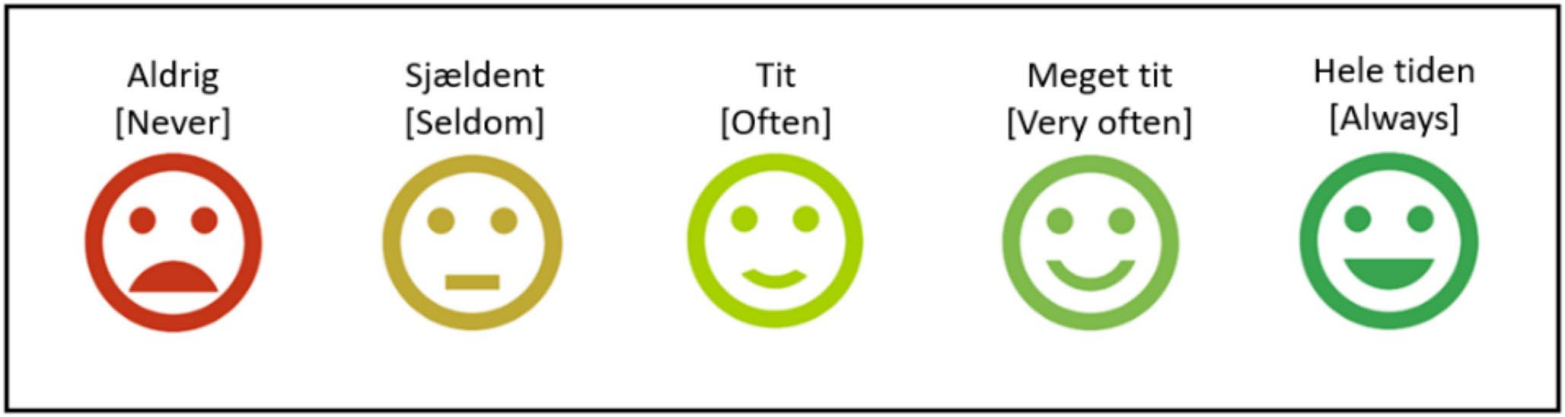
Illustration of the graphical 5-point likert scale

The development process followed four steps: (1) Preparation of script, (2) preparation of visuals, (3) recording of audio, and (4) synchronization with video (PowerPoint slideshow with audio). First, a script with an introduction, the specific questionnaire questions, response-scales, descriptions of the illustrations, and the smiley-face scales was written in order to create clarity and simplicity in language and to ensure alignment between the audio narration and the graphic illustrations. This script was then used to design PowerPoint slides comprising of a short introduction slide and a slide for each questionnaire item (question and response categories) with simple graphic illustrations and smiley-face supported scales. The introduction slide included a brief explanation of the answering process with simple visuals demonstrating how to play the video, the answer scale, and how to proceed to the next question. Each questionnaire item featured one slide, with the question appearing as text, simple graphics, and the text of the response scale (5-point Likert scale) which was supplemented with colored smiley-faces (see Fig. 1). Research show that smiley-face supported scales can help children from the age of seven grasp the emotions or experiences being assessed, making it easier for them to respond accurately [40, 41]. In Denmark, red, yellow, and green colors are widely used and recognized symbols of emotional or experiential intensities, ranging from positive (green) to negative (red). Thus, the colored smileys were chosen to reinforce the emotional valence or experiential intensity of each response. Both the selected smileys and colors have been found to be age-appropriate for Danish children in similar questionnaires [38, 39].

The purpose of the slides was to create visuals that complemented and aided the self-reporting process of the questionnaire, using clear fonts, colors, and simple graphics to enhance readability, along with the audio narration. Next, the audios for each question and scales were recorded following the script, utilizing the ‘Memoer’ app on iPhone.

Efforts were directed toward maintaining a consistent tone and pace to enhance listener comprehension, thereby reducing cognitive load, and enabling focused attention on the content rather than distractions caused by variations in delivery. Key points were highlighted through the deliberate use of vocal inflections. The recordings from ‘Memoer’ were then uploaded and inserted into the PowerPoint slides. The timing and order of the audio and text were adjusted utilizing the feature ‘Animations’ to obtain coherence between audio, text, and visuals on each slide. A preview of the presentation was used to confirm that audio and visual components aligned precisely. Finally, the PowerPoint slideshow with audios for the introduction and each question was exported to MPEG-4 video format and uploaded on a private YouTube channel. The videos were embedded in the electronic questionnaire; each page displayed a video including the respective question and the response scale located below the video. The format can be administered through any platform that supports embedded videos. In this study, the format was set up in the electronic survey program SurveyXact (Rambøll Management, Consulting version 6.10, Copenhagen, Denmark). Illustrations of selected items of the video-assisted version are enclosed in Appendix 1.

### Phase 2: pre-testing and cognitive interviewing

In order to further adapt and optimize the age-adapted language and video questionnaire it was pilot-tested by two researchers (MK and PM), on 44 children from 1st and 2nd grade (seven to nine years of age) involved in a pilot study on the health effects of the multicomponent ‘Generation Healthy Kids’ intervention in early-January 2023. Prior to the test, written consent was obtained from the legal guardians of 44 out of 81 children across four classes (54%) [42]. The aim was to assess whether the age-adapted and video-assisted version was relevant, reasonable, unambiguous, and clear to the target group and whether further adjustments were needed to aid the children’s understanding [43]. This process involved two steps. First, we noted which questions, response options, and/or issues related to the video-format prompted children to raise their hands for assistance. Next, we conducted cognitive, informal debriefing interviews to gather feedback and comments from the children regarding the questions, response options, and/or issues related to the video format that had caused difficulties. During the interviews, alternative wordings were explored and discussed with the children. To ensure consistency in our approach, we used a semi-structured format to conduct the interviews (see Appendix 2) and reported the observations made, the experiences encountered, and the overall insights gleaned during the pre-testing. Since the KIDSCREEN manual and the original developers, to our knowledge, have not explicitly provided detailed definitions of the envisioned meaning for each item, the analysis of these interviews aimed to determine the participants’ interpretation of the items. The modifications made based on the interviews thus reflect the project group’s interpretations of the intended meaning of the items, which were based on the descriptions of the wellbeing dimension each item is intended to measure and has previously been shown to measure in the English version of KIDSCREEN-27, as well as our knowledge of the Danish language. Please, see our condensed and summarized field notes in Appendix 3.

Based on this feedback, the project group made the following small adjustments in wording and format. Many of the children were unsure of the meaning of the word lonely (‘*ensom*’) in item 11 and when explained they understood it as the same as feeling alone. In the Danish language, the word ‘alone’ (‘*alene*’) is more commonly used and well-known within this younger age group, whereas the word or concept lonely seemed difficult to grasp. Thus, item 11, “Have you felt lonely (*‘Har du følt dig ensom’*)?” was changed to “Have you felt lonely or alone (‘*Har du følt ensom eller alene*’)?”. Also, items 16, 18, and 19 were difficult for the children to comprehend. The difficulties were related both to the wording of the items and the constructs themselves, which were challenging for the children to understand and relate to, likely due to their age. Particularly, the items 18 and 19 concerning having the money they need were difficult for the children to relate to, as they do not yet have their own money. While attempts were made by the researcher to verbally simplify and age-adjust the wording, the explanations were not sufficient to resolve the children’s questions or misinterpretations of the items. Therefore, the wording of these three items was retained for further confirming or challenging of our experiences on a larger sample.

### Phase 3: investigation of the psychometric properties

**Fig. 2:**
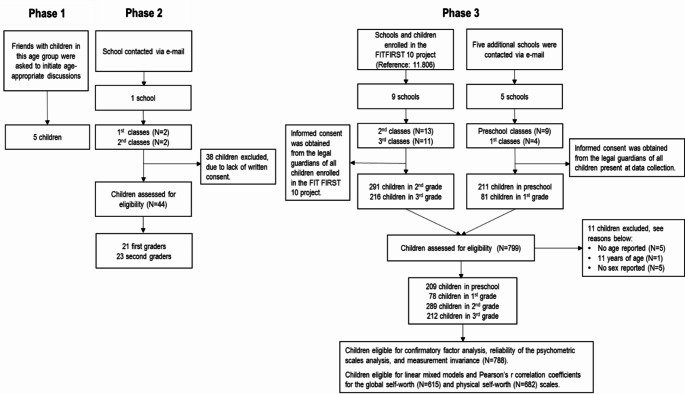
Sampling flowchart for the selection of participants in phase 3

Prior to data collection, informed consent was obtained from the legal guardians; a total of 799 children were assessed for eligibility. See Fig. 2 for the sampling flowchart depicting the selection of participants in phase 3.

#### Informal feedback and item reduction

When administering the adjusted instrument to the large sample of children, we again experienced that most children had difficulties understanding and answering items 16, 18 and 19 from the Autonomy and Parent Relation subscale. Therefore, we decided to exclude the three items from the psychometric analyses. For item 16, the phrase “Have your parent(s) treated you fairly” was difficult for participants, across all ages and schools, to understand as intended, and using alternative words (suitable synonyms) provided verbally by the researcher (i.e., in a verbal explanation of the item or explained through a real-world example) did not seem to improve their comprehension of the item. Clarifying the word or concept ‘fairly and unfairly’ by placing it within a sports context seemed to help the children better interpret ‘fairly’. However, they were still unable to grasp its meaning in the contexts of family and parent-child relations. For this reason, the item was not considered age-appropriate for the measure of the intended construct. For items 18 and 19, regarding having the money they need, several children across ages and schools voiced their confusion about the questions during data collection, because they did not yet have their own pocket money, savings, or responsibility for paying their own expenses. Thus, neither of these items seemed age-appropriate. In parent proxy-report, these items will probably make more sense. Consequently, the subscale Autonomy and Parent Relation was reduced from seven to four items and the KIDSCREEN-27 questionnaire was reduced to 24 items. This final version of the Danish age-adapted and video-assisted version of the KIDSCREEN-27 self-report will be referred to as KIDSCREEN-VIDEO.

### Other measures: global and physical self-worth

The global and physical self-worth scales, two subscales theoretically linked to wellbeing, from the Physical Self-Inventory Scale (PSI-S) [44], each with a one-week recall period, were used to investigate convergent validity. The global self-worth (GSW) subscale is measured by the items “I like myself” and “I want to stay as I am”. The physical self-worth (PSW) subscale is measured by the items “I am happy with myself and what I can do with my body” and “I am happy about all the things I can do with my body” [44]. Each item was answered on a 6-point response scale with an additional option to choose “I do not understand/I do not know”, illustrated by graphical faces (ranging from a very happy to a very unhappy face) [44]. To align with the format used in KIDSCREEN-VIDEO, the PSI-S questionnaire was converted to a video-assisted format.

### Ethical considerations

Study procedures were assessed and approved by the ethics committee ‘Videnskabsetisk Komitée’ (Reference: S-20220094). Study procedures were further registered and approved by The Capital Region’s center for data reviews ‘Videnscenter for Dataanmeldelser’ (Reference: P-2023-70) and by Research & Innovation Organisation (RIO), Legal Services, University of Southern Denmark (Reference: 11.806). Information about the survey was provided to schoolteachers and parents/guardians prior to data collection. Children had the option to withdraw from the project upon request. Informed consent was obtained from the legal guardians of all children in phases 1 and 3. In phase 2, written consent was specifically obtained from all legal guardians, as this phase involved the collection of a blood sample. Please, see the flowchart Fig. 2.

### Data analysis

All scales were scored by calculating the mean scores of its items (sum/number of items). The scores for each subscale were then standardized to a range from zero to one, with one indicating the highest possible score. This scoring approach was applied to make the interpretation of the linear mixed model meaningful. To test and improve data integrity, cases who completed the questionnaire at an unusually fast pace defined as less than 10 seconds per item on average will be identified as this indicates potential inattentiveness [45]. Furthermore, inconsistent responses will be identified using crosstabulation to identify conflicting responses, in e.g., the responses to “Have your parent(s) had enough time for you?” (Item 15) and “Have you been able to talk to your parent(s) when you wanted to?” (Item 17)”.

### Investigation of internal structure

The internal structure of the questionnaire was examined by a confirmatory factor analysis (CFA) using the RStudio 2023.06 package, lavaan [46] and treating the data as ordinal. CFA was done on all participants with complete data (*N* = 788). To inspect the model fit indices, the following cut-off criteria suggesting an acceptable model fit, were used: Chi-square/df < 5.00, comparative fit index (CFI > 0.95), Tucker-Lewis index (TLI > 0.95), root mean square error of approximation (RMSEA < 0.06), and root mean square residual (SRMR < 0.08) [47, 48]. In this study, factor loadings ≥ 0.60 were considered satisfactory, loadings between 0.30 and 0.59 were carefully interpreted in the context of the other internal validity measures to determine whether the item could be deemed reasonable [49, 50]. Loadings < 0.30 was considered insufficient [49–51]. The RStudio code for the CFA can be provided upon request.

### Reliability of the psychometric scales

Reliability measures were computed in SPSS Statistics 23 (IBM Corp, Armonk, NY, USA) and analyses were done on all participants (*N* = 788). The internal consistency of each subscale of wellbeing and HRQoL was estimated using the Cronbach’s alpha and McDonald’s omega coefficient [52]. Values of 0.7 or higher were considered acceptable [53]. However, for scales with fewer than five items, (i.e. the three dimensions: Autonomy and Parent Relation, Social Support, and School Environment) values above 0.6 were considered acceptable [54].

### Investigation of the associations with other variables

As wellbeing and self-worth was hypothesized to be positively related, positive associations between these variables was used as indicators of convergent validity of the KIDSCREEN-VIDEO. The association was analyzed using linear mixed models with the five subscales of wellbeing and HRQoL as dependent variables, and the GSW and PSW subscales as predictors adjusting for sex and age, as well as the nesting/clustering of the data on classes and schools. The associations between the subdimensions of KIDSCREEN and other variables were evaluated using data from all participants with complete responses on both the GSW and PSW subscales. Children who answered, “I do not understand/I do not know” to one or both items in either subscale were excluded from these analyses. These analyses were conducted using R Studio version 2023.06 packages, lme4 and lmerTest. Convergent validity was also evaluated using Pearson’s r correlation coefficients for the unadjusted associations between the KIDSCREEN-VIDEO wellbeing measures and the self-worth measures. Pearson’s r coefficients < 0.1 were considered trivial, 0.1–0.29 were considered small, 0.30–0.49 as moderate, and ≥ 0.5 as high [55]. All significance tests were two-tailed and P-values below 0.05 were considered statistically significant.

### Measurement invariance

Measurement invariance testing was conducted to assess whether the factor structure of KIDSCREEN-VIDEO was consistent across sex and age using the RStudio 2023.06 package, lavaan [46]. The two age groups compared were children aged six to seven years (*N* = 254), and children aged eight to ten years (*N* = 534). Model fit indices for three levels of measurement invariance were assessed and compared. The configural model examined equivalence of model form i.e., whether the same pattern of fixed and free factor loadings was specified across groups. The metric model examined equivalence of factor loadings across groups. The scalar model examined equivalence of factor loadings and item thresholds across groups. An adequate configural invariance indicates a similar factor structure across groups, allowing for potential differences in the factor structure across groups. A decrease in model fit between consecutive levels of invariance suggests that the respective invariance assumption does not hold. To inspect measurement invariance, the following cut-off criteria was used: Comparative fit index (ΔCFI < 0.01), Tucker-Lewis index (ΔTLI < 0.01), root mean square error of approximation (ΔRMSEA < 0.015), and root mean square residual (ΔSRMR < 0.01). The RStudio code for the measurement invariance can be provided upon request.

## Results

### Participants and recruitment

The adjusted instrument was administered to 799 children, across 14 schools, across nine municipalities. The participants were recruited through cooperation with municipalities and schools of the Regions South and Zealand, Denmark using a convenience sampling procedure. Inclusion criteria were all children from preschool to third grades (age six to ten years old). If no age or sex was reported, participants was excluded (*N* = 11), see Fig. 2. The data set was complete with no missing answers on any of the KIDSCREEN items, allowing for analyses to be performed on the entire data set. The 788 participants were equally distributed on sex (49.8% were girls) and were between six and ten years of age. In total, 26.5% of the children were in preschool classes (age 6–7), 10.0% were first graders (age 7–8), 36.6% were second graders (age 8–9), and 26.9% were third graders (age 9–10) (overall mean = 7.9; SD = 1.08; range 6.1–10.8 yrs.). The data were collected from mid-January 2023 to June 2023. The completion time ranged from seven minutes to 52 min (M = 19.84; SD = 6.67) i.e., the fast completers, on average, used 15 s per item and crosstabulations of potentially conflicting responses indicated data consistency (See Table 1).

**Table 1:** Sex and grade distribution of study participants

	*N* (%)
**Females**	392 (49.8)
**Total children**	788 (100)
Preschoolers	209 (26.5)
1st graders	79 (10.0)
2nd graders	288 (36.6)
3rd graders	212 (26.9)

### Mean scores and investigation of internal structure

The mean scores, standard deviations, skewness, and kurtosis for all scales and variables are presented in Table 2 As reported, study variables were normally distributed, as skewness and kurtosis values fall between − 1.02 and 1.90 [56], and all scores ranged between 0 as the minimum and 1 as the maximum. Table 2 also reports low item intraclass correlations, between 0.013 and 0.043, indicating a low impact of the nesting structure.

**Fig. 3:**
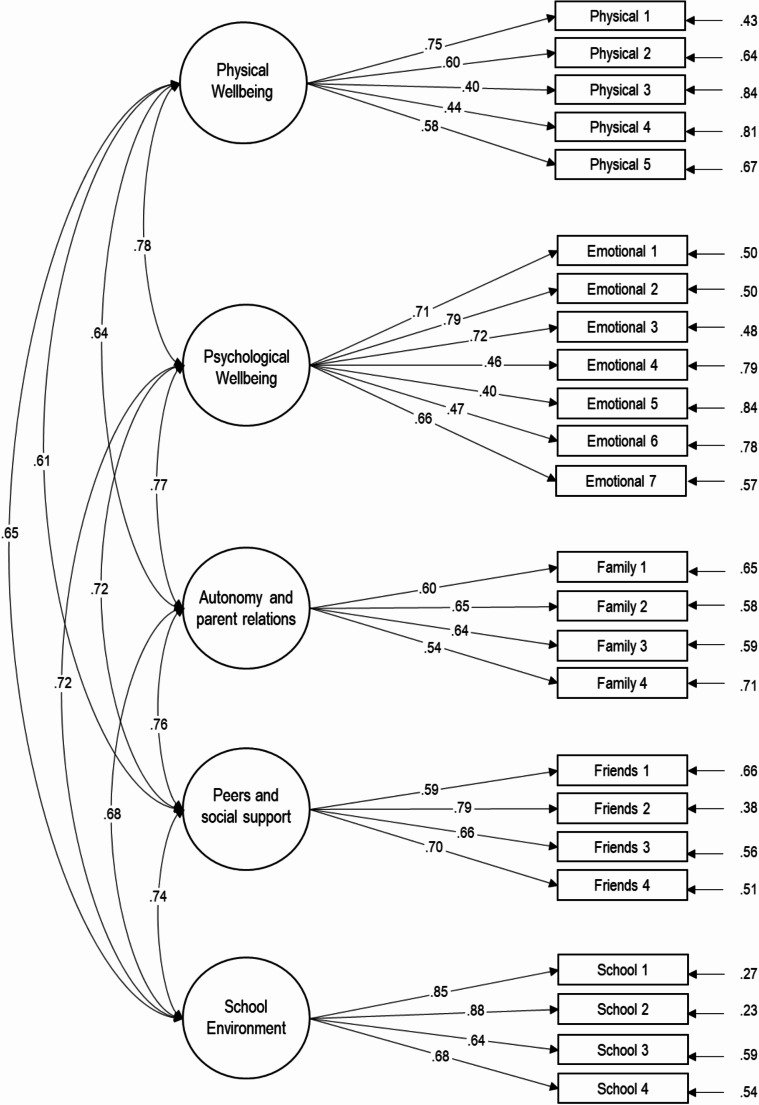
Confirmatory factor analysis showing significant standardized factor loading, significant factor correlations and significant error term correlations. Note: *N* = 788

CFA was done on all eligible participants (*N* = 788). The overall model fit for the five-factor model showed a statistically significant chi-square test (*X*^2^/df = 724.586/242). Initial CFA revealed an acceptable model fit. The model fit indices were as follows: CFI = 0.983; TLI = 0.980; RMSEA = 0.050 [90% CI 0.046-0.055]; SRMR = 0.050. Out of the 24 factor loadings, 16 were considered satisfactory, ranging from 0.60 to 0.88. However, eight loadings fell between 0.40 and 0.59, prompting discussions about the internal structure and age-appropriateness of certain items. The factor loadings, covariances, and error terms are illustrated and reported in Fig. 3.

### Reliability of the psychometric scales

The internal consistency of the scales was evaluated with Cronbach’s alpha and McDonald’s omega [52, 53] and are presented in Table 2. The reliability coefficients for four of the five dimensions of wellbeing and HRQoL, including Psychological Wellbeing, Autonomy and Parent Relation, Peers and Social Support, and School Environment, were acceptable to good, with Cronbach’s alpha values ranging from 0.65 to 0.80. For the Physical Wellbeing scale, internal reliability coefficients were just below acceptable with a Cronbach’s alpha value of 0.65 and Omega of 0.64.

**Table 2:** Sample descriptive and scale reliability

	Mean (SD)	Skewness	Kurtosis	α	Ω	ICC
Physical Wellbeing (5 items)	0.67 (0.18)	-0.425	0.036	0.651	0.644	0.043
Psychological Wellbeing (7 items)	0.74 (0.15)	-1.018	1.902	0.738	0.724	0.024
Autonomy and Parent Relations (4 items)	0.68 (0.19)	-0.563	0.153	0.650	0.658	0.043
Peers and Social Support (4 items)	0.75 (0.19)	-0.903	0.903	0.728	0.726	0.013
School Environment (4 items)	0.73 (0.21)	-0.891	0.603	0.797	0.801	0.029

Note. *N* = 788. Standard deviation, SD; Cronbach’s alpha, α; Omega, Ω; Inter-class correlation coefficient, ICC

### Investigation of the associations with other variables

The associations between the five subdimensions of wellbeing and HRQoL and other variables were evaluated using data from all participants with complete responses on the GSW (*N* = 615) and PSW (*N* = 682) subscales. The 12 linear mixed models adjusted for sex and age, showed a positive and significant relationship between self-worth and the five subdimensions of HRQoL and wellbeing, with e.g., a 10% increase in wellbeing being associated with a 4.1% increase in global self-worth (see Table 3). Additionally, the scores for the five subdimensions displayed moderate to high positive correlations with both global- and physical self-worth, with correlations ranging from 0.30 to 0.58, see Table 4.

**Table 3:** Twelve linear mixed models adjusted for sex, age, and cluster

	*N*	β (SE)	
**Physical Wellbeing**			
Global Self-worth		615	0.29 (0.03) *
Physical Self-worth		682	0.33 (0.03) *
**Psychological Wellbeing**			
Global Self-worth		615	0.41 (0.03) *
Physical Self-worth		682	0.37 (0.03) *
**Autonomy and Parent Relations**			
Global Self-worth		615	0.40 (0.03) *
Physical Self-worth		682	0.44 (0.03) *
**Peers and Social Support**			
Global Self-worth		615	0.49 (0.04) *
Physical Self-worth		682	0.43 (0.03) *
**School Environment**			
Global Self-worth		615	0.49 (0.04) *
Physical Self-worth		682	0.52 (0.04) *

Note. Unstandardized beta values, β; Standard error, ER; p-values below 0.001*

**Table 4:** Variable intercorrelation matrix (Pearson’s *R*)

	*N*	1	2	3	4	5	6	7		
1. Physical Wellbeing		788	1							
2. Psychological Wellbeing		788	0.487	1						
3. Autonomy and Parent Relations		788	0.411	0.520	1					
4. Peers and Social Support		788	0.399	0.513	0.528	1				
5. School Environment		788	0.426	0.549	0.490	0.571	1			
6. Global Self-worth		615	0.302	0.465	0.399	0.421	0.571	1		
7. Physical Self-worth		682	0.346	0.440	0.428	0.415	0.421	0.582	1	

### Measurement invariance

The measurement invariance results are presented in Table 5. The results show no significant differences in model fit indices from the original CFA model (*X*^2^/df = 724.586/242; CFI = 0.983; TLI = 0.980; RMSEA = 0.050 [90% CI 0.046-0.055]; SRMR = 0.050), indicating the overall model fit for the five-factor model was consistent across sex and age groups. As seen in Table 5, both the configural, metric, and scalar model showed a comparable model fit indicating metric and scalar invariance across different age groups and sex. This suggests that the relationships between the five dimensions and their items, as well as the starting points of the measurement scales, were equivalent across both sex and age groups.

**Table 5:** Measurement invariance across sex and age groups. Differences between the original CFA model and the configural, metric, and scalar models, respectively

	ΔCFI	ΔTLI	ΔRMSEA	ΔSRMR	
**Configural invariance**					
Sex		< 0.001	< 0.001	< 0.001	< 0.001
Age		< 0.001	< 0.001	< 0.001	< 0.001
**Metric invariance**					
Sex		-0.003	-0.002	0.004	0.012
Age		-0.004	-0.003	0.006	0.012
**Scalar invariance**					
Sex		-0.003	0.001	0.001	0.008
Age		-0.002	0.001	0.001	0.010

Note. *N* = 788. CFA, confirmatory factor analysis. Age groups: (a) below eight years of age and (b) above eight years of age. Change in comparative fit index, ΔCFI; change in Tucker–Lewis index, ΔTLI; change in root mean square error of approximation, ΔRMSEA; change in root mean square residual, ΔSRMR; Chi-square, *X*^2^; degrees of freedom, df

## Discussion

This study addresses an important gap in the questionnaire instruments available for self-report of subjective wellbeing among healthy children aged six to ten years. By developing a video-assisted format and using an age-adapted version of KIDSCREEN-27 self-report (the KIDSCREEN-VIDEO) we were able to collect self-reported measures of wellbeing and HRQoL among Danish children aged six to ten which overall showed satisfactory indicators of validity and reliability.

The CFA showed an acceptable, approaching good, model fit supporting the five-dimensional structure of the questionnaire, and item loadings were generally satisfactory, ranging from 0.60 to 0.88, also supporting the validity of the psychometric scales; however, some loadings fell between 0.40 and 0.59. The linear mixed model analyses, and Pearson’s r correlation coefficients showed positive associations between the KIDSCREEN-VIDEO measures of wellbeing and both self-worth scales, thereby supporting their interrelationship. The combination of the fit indices and overall indications of validity supports that the KIDSCEEN-VIDEO is a valid self-reported measure of wellbeing for Danish children aged six to ten.

These results are consistent with CFAs reported in the original KIDSCREEN project validation studies on children aged eight to 18, across 13 European countries [19], a study on Norwegian ten-year-old children [57], and Chilean adolescents [58], as well as for a clinical sample of three European (Austria, Germany, and Switzerland) children and adolescents aged eight to 18 years [59]. Results also indicate that the use of KIDSCREEN-VIDEO among children aged six to ten might provide more precise predictions compared to, for example, administering KIDSCREEN-27 to Swedish children aged eight to 11 (RMSEA = 0.074 [90% CI 072-0.076]; CFI = 0.963) [60] and the self-reported version administered to Romanian children aged six years (RMSEA = 0.072 [NR]; CFI = 0.847). Hence, based on our results, the KIDSCREEN-VIDEO provides a promising self-reported measure for wellbeing within this age-group.

The linear mixed model analyses, and correlation coefficients showed a moderate to high positive relationship between the two self-worth scales and both the overall wellbeing and HRQoL score as well as the five dimensions. This indicates convergent validity of KIDSCREEN-VIDEO. The reason for the decision to include only two subscales with a total of four items for assessment of the convergent validity was based on the necessity to keep an age-appropriate length of the questionnaire for children as young as six years. By focusing on a smaller subset of four items, the assessment became more targeted and less burdensome for the children. Thus, the decision ensures that the questionnaire remains accessible and feasible for use in this age group.

The reliability analysis indicated acceptable to good internal consistency for four out of five dimensions, while the values for the Physical Wellbeing dimension only closely approached the cut-off for acceptable consistency. The main shortcoming of the KIDSCREEN-VIDEO regarding internal reliability therefore seems to be for the Physical Wellbeing scale, suggesting further consideration or improvement of items in this domain (i.e., items 1–5). The original KIDSCREEN project showed that the lowest level of scaling success was found for item 1, “How would you say your health is?”, of the Physical Wellbeing dimension [19]. Therefore, improving this item alone might enhance the results. In KIDSCREEN-VIDEO, this item was rephrased as “How are you feeling/doing?”. However, the results suggest that further refinement may still be necessary.

The Cronbach’s alpha values of this study (ranging from 0.65 to 0.89) are slightly higher than those found in the self-reported version administered to Romanian children aged six years (ranging from 0.56 to 0.78) [28]. However, the coefficients are lower than the Cronbach’s alpha values reported in the original KIDSCREEN project on children aged eight to 18 (ranging from 0.80 to 0.84) [11]. In a study on Norwegian children aged ten years, the Cronbach’s alpha values ranged from 0.73 to 0.83 [57], thus, our result’s approximate similar reliability to those reported in the literature for a population sharing similar characteristics in terms of culture, but with a higher mean age.

The reliability of children as informants of their own wellbeing, using different HRQoL instruments, have been supported in the literature [22, 23]. Measures of young children’s self-reported wellbeing, such as the KIDSCREEN-VIDEO, are important to develop and use, because proxy-report of children’s wellbeing, (e.g., parents answering on behalf of the child), requires the reporter to be present in all the child’s daily contexts and to have knowledge of how the child perceives and feels in these situations. Alternatively, the adult interviews the child without biasing the answers [23, 25]. Yet, parent proxy-report may be useful as a supplemental source of information, when using the KIDSCREEN-VIDEO, as this version no longer includes items relating to financial resources. The Autonomy and Parent Relation dimension is originally intended to measure the quality of the interaction between child and parent as well as whether the child feels loved and supported by the family [11]. The KIDSCREEN-VIDEO excluded two items assessing family support in terms of financial resources, to adapt a questionnaire that was suitable for self-report among children younger than ten years.

Overall, we found that the use of a video-assisted questionnaire format supported and enabled self-reporting of wellbeing among Danish children aged six to ten. Features of the video-assisted format of the questionnaire such as structure, audio, illustrations, and smiley-supported scales appeared age-appropriate. This is in line with the literature that suggests using an electronic format enhances self-reporting among children [23], including those in a Danish population aged seven to 12 years [38, 39]. In all three test and adaption phases a high level of interest, attention, and engagement was observed among the children and that the items and response options were read aloud helped compensate for diversity in reading abilities. Although the age-appropriateness of the colored smiley-assisted response scale was considered, tested, and discussed thoroughly to ensure reliable responses and engagement, some concerns should also be raised [40, 41, 61]. Firstly, the use of colored smileys can potentially lead children to select responses they believe are more socially acceptable, thereby introducing social desirability bias into their answers [41]. Furthermore, the use of colors necessitates consideration of cultural context and emotional associations, as children from different cultures might interpret the color range green, yellow, red differently [41]. On the other hand, research indicates that pictorial response formats can improve understanding and engagement for children [40, 61]. Therefore, alongside age-appropriateness, it is important to consider the cultural and hence contextual appropriateness of the smiley and color illustrations of the response scale, ensuring it aligns with culture specific interpretations and understandings of the children involved [41, 61].

### Strengths and limitations

The study has both strengths and limitations that should be considered when interpreting the results and determining the appropriateness of using the KIDSCREEN-VIDEO in future studies. A major strength of the KIDSREEN-VIDEO is its coverage of five dimensions of wellbeing and HRQoL, as demonstrated in the conceptual and empirical framework of the European KIDSCREEN project [11, 13, 19]. Along with the relatively large sample size, a notable strength of the study is the use of a video-based questionnaire format, which enabled children who were not yet able to read to self-report on their wellbeing. The video-assisted format, which included tablets and headphones, also had the positive side-effect of keeping the children engaged and focused, thereby minimizing distractions and interference while completing the questionnaire. In addition, the video-format required minimal assistance, with one research assistant or teacher successfully helping approximately 24 children fill in the questionnaire; an efficiency that highlights another strength of the KIDSCREEN-VIDEO. However, the video-assisted format require access to resources such as tablets and headphones, and a good internet connection. This could introduce extra costs to, for example research projects, composing a possible limitation. A poor internet connection can cause impatience and interfere with the children’s ability to self-report. Therefore, future studies should consider using a platform that supports embedded videos in an offline format, thus not requiring internet access.

The study used convenience sampling, which is a limitation as this might introduce selection bias. However, the included schools represented a broad range of public schools from both urban and rural areas. Specially, the consent rate in phase 2, where the items were pre-tested and adjusted for interpretation, was low, which introduces the potential for selection bias in this sample. We believe there is a risk that children who have negative feelings or perceptions about participating in biomedical measures, such as blood samples and blood pressure readings, are underrepresented in this phase [42, 62]. Another limitation is that the study is unable to evaluate whether KIDSCREEN-VIDEO demonstrates sensitivity to change, given the cross-sectional study design. Thus, to adequately assess the instrument’s ability to measure changes over time, it should be tested within an experimental or longitudinal study design. Future research should focus on translating and validating the questionnaire in other major languages and evaluate other types of validity and reliability, such as a test-retest reliability of the instrument, the predictive validity. Additionally, since our study targeted a healthy population, we suggest that future studies include more diverse samples to test the robustness and generalizability of the KIDSCREEN-VIDEO across various demographic groups.

The study only analyses measurement invariance across sex and age. Future studies should examine measurement invariance across other background variables, such as socio-economic status and the presence of chronic conditions. It can be considered a major limitation of this version of KIDSCEEN that, to ensure comprehension, relevance, and internal reliability of the Autonomy and Parent Relation scale for children aged six to ten years, three items had to be removed, resulting in a narrower subscale. The item reduction complicates comparisons with studies using the original KIDSCREEN-27 version as well as Rasch measurement analysis with T-scores. However, the scale can be standardized for such comparison for example by calculating the mean score of the four items in the reduced Autonomy and Parent Relation subscale and multiplying by seven [19]. It may be considered even more problematic that the item reduction could result in the scales measuring slightly different elements. The Autonomy and Parent Relation dimension involves the quality of the interaction between child and parent/caregiver as well as whether the child feels loved and supported by the family. It also assesses the individual’s perceived level of autonomy and the perceived quality of the financial resources of the child. Excluding item 18 (“Have you had enough money to do the same things as your friends?”) and item 19 (“Have you had enough money for your expenses?”) omits the aspect of perceived financial resources. Therefore, we recommend that future studies using KIDSCREEN-VIDEO for this age group exclude the financial resources component when describing the content of this dimension, clarifying that this aspect is not included. Another limitation to the construct validity of the Autonomy and Parent Relation scale is the exclusion of item 16 (“Have you parents treated you fairly?”). This item serves as an indicator of both perceived autonomy and support in the child’s relation to their parents. However, it can be argued that feelings of support and autonomy are somewhat addressed by the remaining four items of the subscale, though with less nuances. Furthermore, one should be aware that the cut-offs are based on norm data from children aged eight, so results based on cut-offs should be interpreted with caution, especially among children younger than this age.

### Implications for future research

This study provides evidence to suggest that KIDSCREEN-VIDEO, a video-assisted and age-adjusted version of the KIDSCREEN-27 self-report, may enable a valid measure of self-reported wellbeing and HRQoL among children aged six to ten. In intervention and survey studies, where such measures are needed, it therefore seems to be a valuable selection and important contribution.

Knowing the growing challenges of poor wellbeing among children worldwide, we suggest that future research continues to focus on the measurement and monitoring of wellbeing in this age-group. The Sustainable Development Goals set by WHO aim for better health and wellbeing for all at all ages by 2030 [63]. Thus, early self-report of subjective wellbeing and quality of life can be an important part of the progress toward this target.

For use in other languages and cultures, KIDSCREEN-VIDEO needs to be translated, including the video-assisted format with voice-over, and a revalidation would be ideal. We are currently using the KIDSCREEN-VIDEO in a large, ongoing intervention study for children aged seven to 11 [62], and can confirm that the format can be administered through any platform that supports embedded videos; in this case, REDCap.

## Conclusion

This study contributes to the scarce literature on self-reported measures of wellbeing among children aged six to ten. The KIDSCREEN-VIDEO is an age-adapted and video-assisted version of KIDSCREEN-27 for collecting self-reported measures of HRQoL and wellbeing among Danish children aged six to ten. The psychometric evaluation of the KIDSCREEN-video questionnaire showed good psychometric properties in terms of model fit, primarily satisfactory loadings on the five intended dimensions, and convergent validity. Except for the Physical Wellbeing dimension, the internal reliability of the scales was also satisfactory. This suggests that the KIDSCREEN-VIDEO enables measures of self-reported wellbeing among children aged six to ten. In studies where it is important, to measure and monitor the subjective feelings of wellbeing among children too young to read the KIDSCREEN-VIDEO seems a promising tool. However, in many cases language translations and revalidations would be needed. Furthermore, it is important to be aware that perceived financial resources could not be included in the Autonomy and Parent Relation scale of KIDSCREEN-VIDEO, which narrows the content of this scale.

## Electronic supplementary material

Below is the link to the electronic supplementary material.

Supplementary Material 1

Supplementary Material 2
