# Supplementary material for: Enabling six- to ten-year-old children to self-report their wellbeing and quality of life: development and psychometric investigation of an age-adapted and video-assisted version of the KIDSCREEN-27
Source: Qual Life Res. 2025 Mar 1;34(6):1599–614. doi: 10.1007/s11136-025-03939-6 (PMC12119647; doi:10.1007/s11136-025-03939-6)
Supplement: Supplementary file 1 — Supplementary Material 1 [file 11136_2025_3939_MOESM1_ESM.pdf]

Appendix 1. Illustrations of selected items/pages of the video-assisted version

An example of a video/page can be watched with the following link: https://youtu.be/9GiafLardrU

Hvordan har du det?

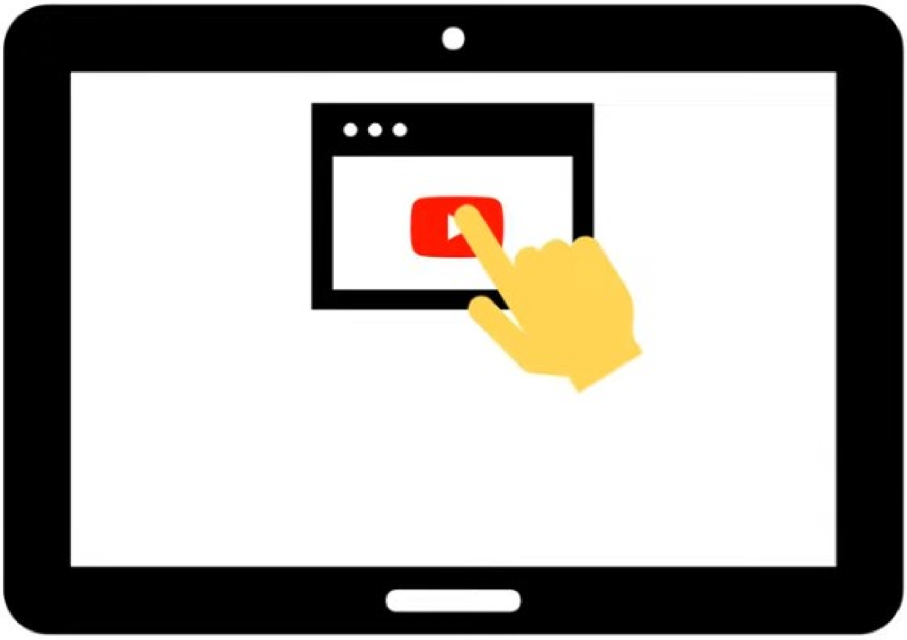

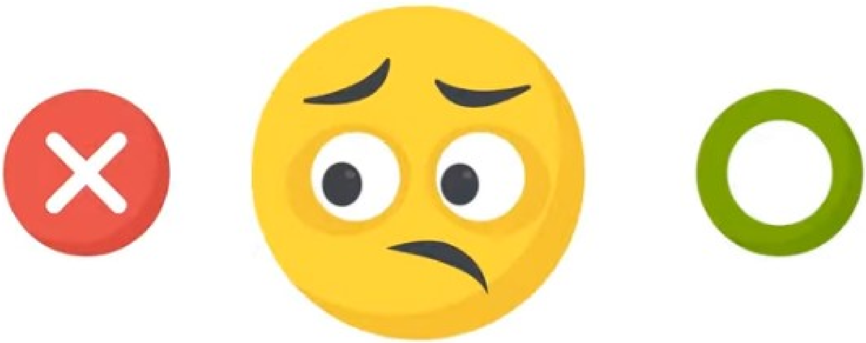

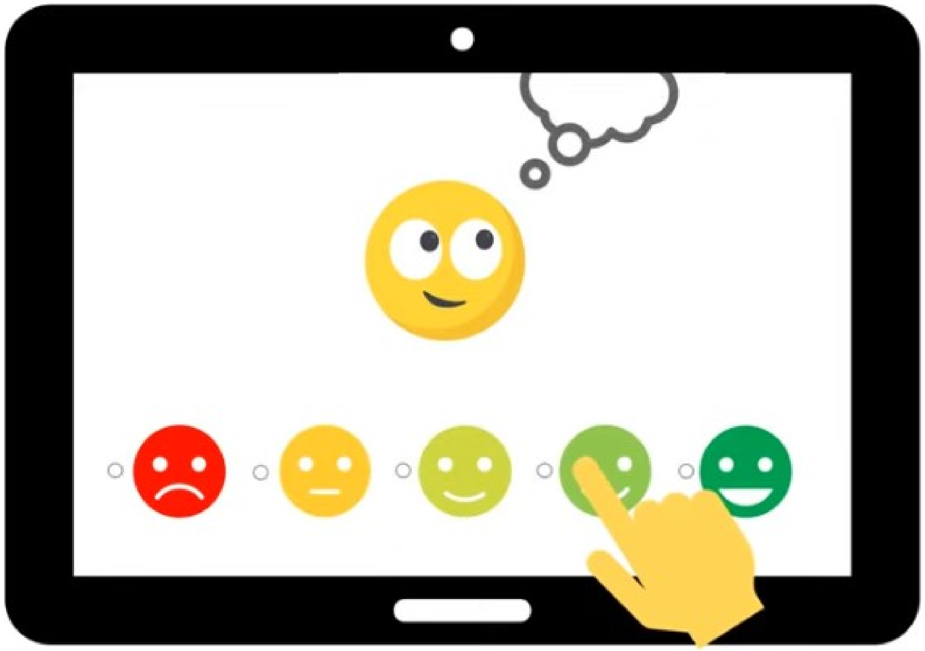

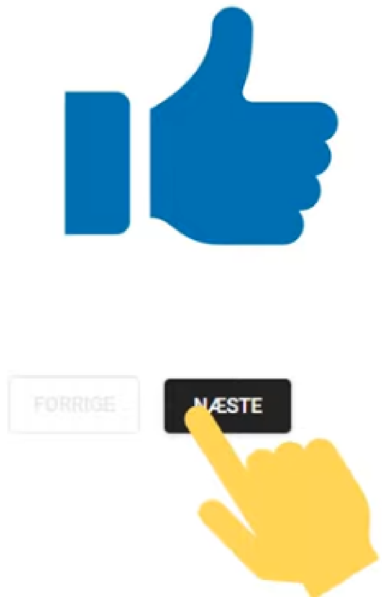
FORRIGE
NÆSTE

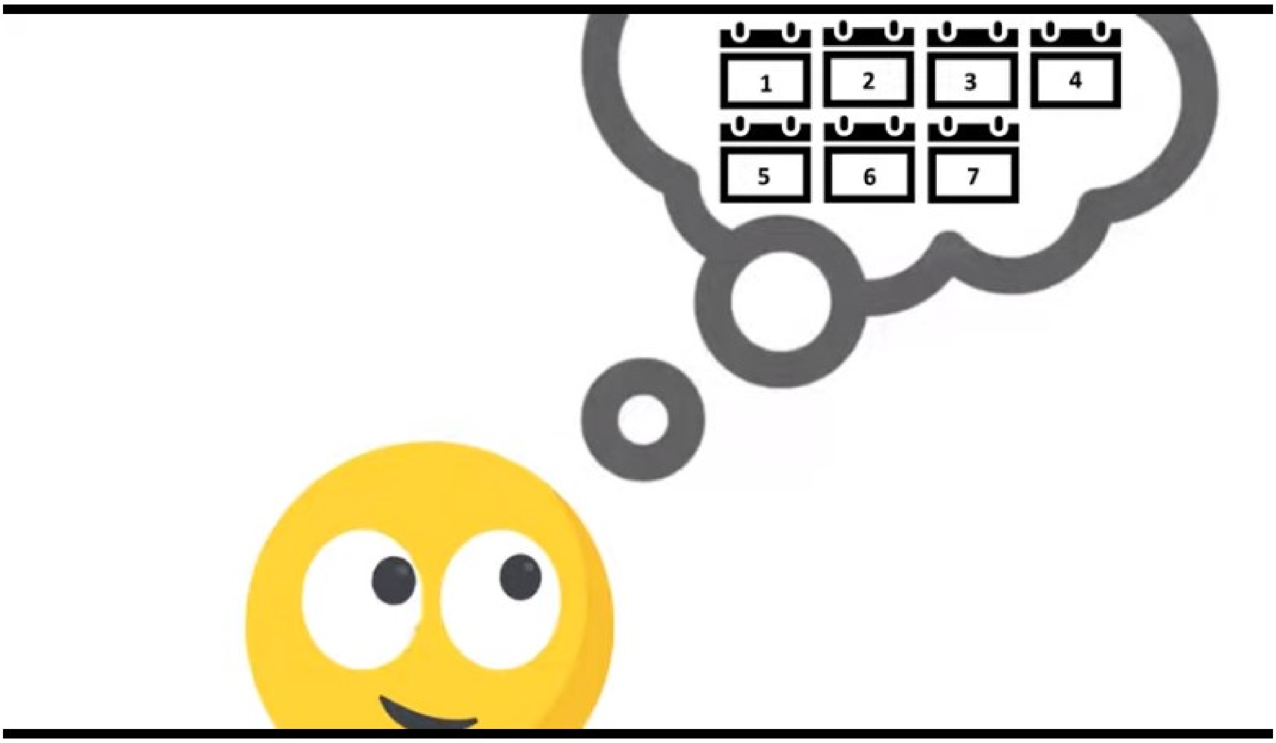

3

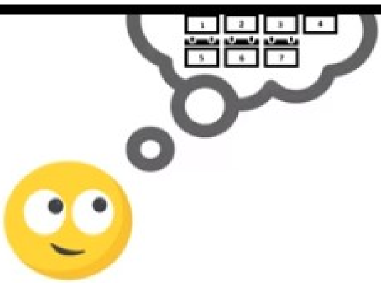

Har du været aktiv, fx løbet, klatret og cyklet?

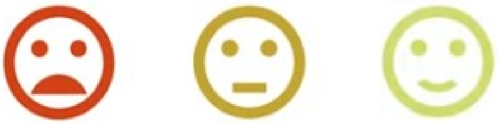

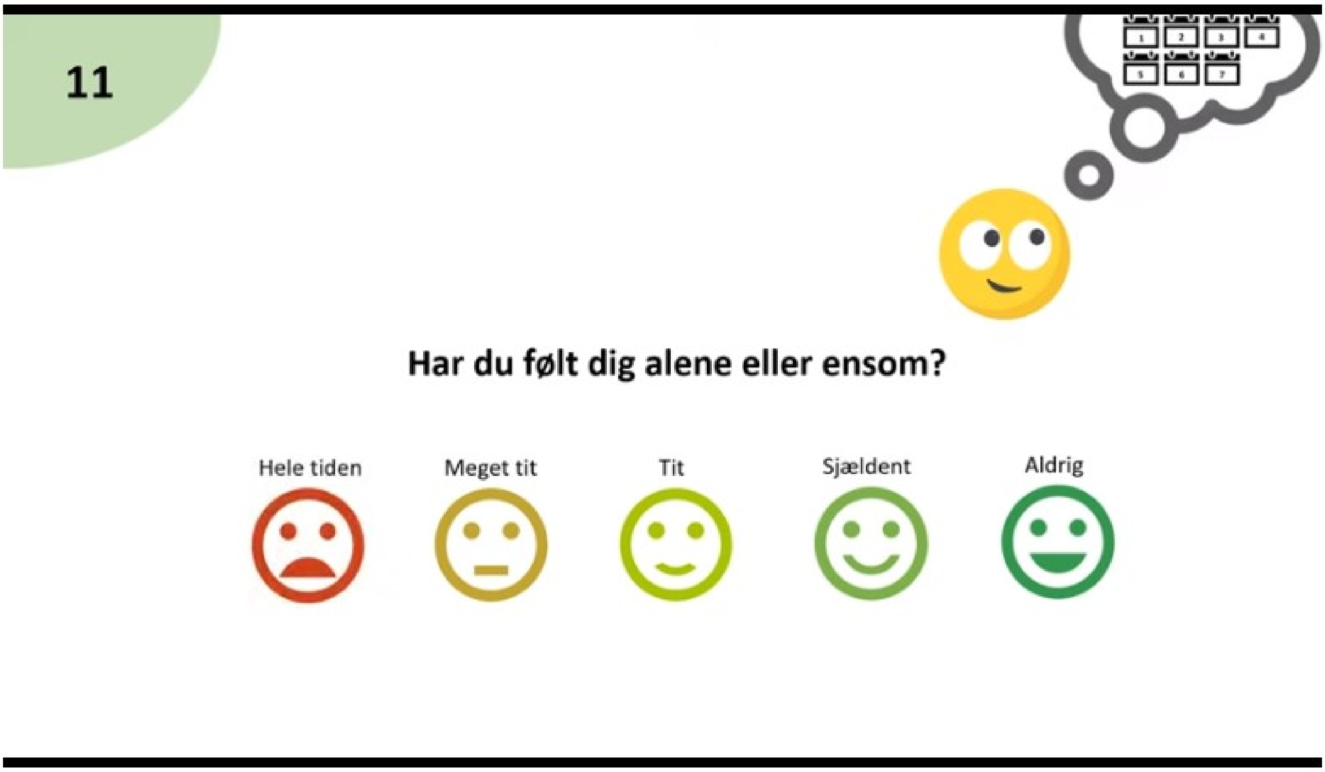
11
Har du følt dig alene eller ensom?
Hele tiden
Meget tit
Tit
Sjældent
Aldrig

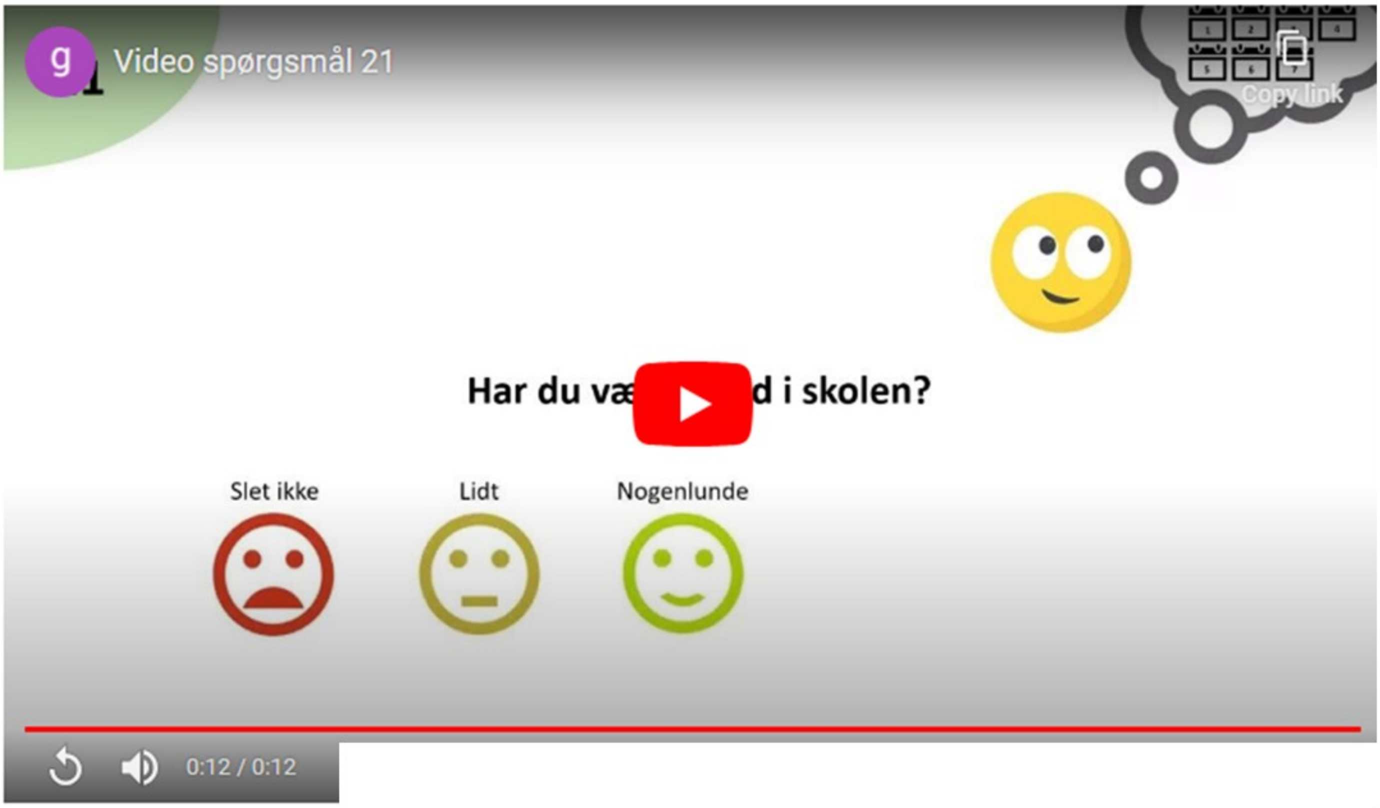
Video spørgsmål 21
Copy link
Har du væ
d i skolen?
Slet ikke
Lidt
Nogenlunde
0:12 / 0:12

Appendix 2. Interview guide

Interview guide for children's experiences with answering the video-assisted questionnaire

1. *Overall impressions*
    - What did you think about the questionnaire?
    - Was there anything you liked or didn't like about it?

2. *Length of the questionnaire*
    - How did you feel about the number of questions in the questionnaire?
    - Did you find it easy to stay focused while answering the questions? Were there any points where you felt tired or bored?

3. *Format/structure*
    - How did you feel about the video-assisted format? Did it help you to understand the questions and answers better?
    - Where there any parts of the video that were especially confusing or helpful?
    - Was it easy to understand and follow the questionnaire? Were there any issues with scrolling or clicking on the answers? Did you find it easy to move from one question to next?

4. *Specific items*
    - Were there any questions that you found difficult to understand?
    - For example, how did you understand the question: "Have you had enough money to do the same things as your friends?" (Item 18)
    - How about: "Have you had enough money for the things you needed to buy in everyday life?" (Item 19)
    - And "Can you give me an example of a time when you felt your parent(s) treated you fairly?" (Item 16)

5. *Final thoughts*
    - Is there anything else you would like to share about your experience with answering the questionnaire?

Appendix 3. Measurement of wellbeing and health-related quality – Field Experiences

Length of the questionnaire

Based on our experiences in the classrooms and discussions with the teachers, we believe that it is important to keep as short as possible. Our experience suggests that the quality of responses and the concentration among children can be improved by reducing the number of questions. We observed that the children' concentration decreased over time – and once one or more children had finished, the concentration further decreased among a larger portion of the other children – some began to 'rush' to finish, which may affect the quality of their responses.

Overall experience with individual questions

In general, we found that the children could understand and answer most of the questions independently and without problems/difficulties. There were only three questions that we found particularly difficult for all children across the schools to understand and answer – this observation was based on the number of children raising their hands and asking for assistance to answer them.

16. Have your parents treated you fairly?
18. Have you had enough money to do the same things as your friends?
19. Have you had enough money for the things you needed to buy in everyday life?

Question 16: We found that many children had difficulty understanding the word 'fairly'. We also found it difficult to explain – all present adults (including ourselves) tried in various ways and with words like 'fair', 'proper', 'okay', 'reasonable', etc. Several of us tried to verbally explain it as 'fair', but the children often did not know what 'fair' meant either – however, they could relate to the word 'unfair', but the cognitive aspect of subsequently understanding that 'fairly' should be understood as the opposite of 'unfair' was difficult and often not possible. The explanations often ended with a student, after various attempts at explanations, saying: 'Oh… so it means whether they

are mean to me? Or often scold me? Or treat me badly?'. Additionally, there were also problems with placing the word 'fair' or 'unfair' in a parental context – the children referred to it more as something they could recognize in sports, games, recess, or other social contexts.

Questions 18-19: We found that several children had difficulty relating to 'having money' and 'buying things in everyday life'. For example, several children said, 'but I don't have any money', 'I don't really understand it… because I don't have any money or a job…', 'you can't buy things without money' or 'it's my parents who buy things'. We also found that the context was difficult for the children to relate to, as the question referred to whether they had money; for example, several said 'my parents don't have any/a lot of money' or 'at my grandparents'/parents' house, I always get everything I want'. We did not find that they possessed money themselves or bought things on their own, without the help of their parents or other adults. It is our general impression that children in this age group do not receive pocket money or 'possess' their own money, which our experiences in the classrooms supported. Additionally, we found ourselves telling the children 'Just press the one in the middle then…', as several had difficulties answering these two questions and sometimes said 'I just don't know what to answer…'.

Questionnaire structure/format

Our immediate impression of the video-assisted form in relation to answering questions was that it worked well in practice. However, we experienced small things that could be adjusted if possible, such as being able to click directly on the smileys and not in the circles below, that the children did not have to 'scroll' down to answer, but could see the entire page, and that the children, after hearing and watching the video, could not see 'YouTube ads' for other videos.

Appendix 4. Overview of age adjustments to the item questions

Item	Original KIDSCREEN-27 items in English and Danish version	Age-adjusted items in Danish for KIDSCREEN-VIDEO	English explanation of Danish adjustments – lost in translation
1	ENG: In general, how would you say your health is?<br>DA: Hvordan er dit helbred generelt?	DA: Hvordan har du det?	The change in wording shifts the focus from a specific inquiry about health, prompting the children to think about any health issues, recent illnesses, or general physical conditions, to a more open-ended question about feeling healthy and well – both physically and mentally.
2	ENG: Have you felt fit and well?<br>DA: Har du følt dig rask og godt tilpas?		
3	DA: Har du været aktiv, fx løbet, klatret og cyklet?<br>ENG: Have you been physically active (e.g., running, climbing, biking)?		
4	ENG: Have you been able to run well?<br>DA: Har du kunnet løbe derud af?	DA: Har du kunnet løbe?	The change in wording simplifies the question – directly translated, it becomes "Have you been able to run". Thus, it simply asks if the child has had the ability to run, referring to running in general. In Danish, it implicitly addresses physical capability and fitness, and serves as a casual inquiry about whether they have run recently – including whether they have been able to run well or if they have had difficulties running.<br>The original Danish translation can be difficult to understand, especially for children but also in general, as "derudaf" ('out of it' or 'from there') refers to your running condition, fitness, capacity,

			and physical shape – essentially asking one to make a judgment about it.
5	ENG: Have you felt full of energy?<br>DA: Har du følt dig ful af energi?		
6	ENG: Has your life been enjoyable?<br>DA: Har dit liv været sjovt?		
7	ENG: Have you been in a god mood?<br>DA: Har du været I godt humør?		
8	ENG: Have you had fun?<br>DA: Har du haft det sjovt?		
9.	ENG: Have you felt sad?<br>DA: Har du været ked af det?		
10	ENG: Have you felt so bad that you didn't want to do anything?<br>DA: Har du haft det så dårligt, at du ikke havde lyst til at lave noget som helst?	DA: Har du været så ked af det, at du ikke havde lyst til at lave noget som helst?	The decision to change the translation of "dårligt" ('bad') to "ked af det" ('down') is based on the finding that the children comprehended feeling "dårligt" as meaning being physically ill, we changed "dårligt" to "ked af det" which also means feeling bad but in a more mood related emotional way (feeling low or down).
11.	ENG: Have you felt lonely?<br>DA: Har du følt dig ensom?	DA: Har du følt dig alene eller ensom?	The decision to add the word "alene" (alone) is based on the finding that the children struggled to fully grasp the concept of "ensom" ('lonely').<br>The children were generally more familiar with the experience of being alone, which they interpreted as being lonely, rather than grasping the emotional nuances associated with "ensom" ('lonely'). In contrast, the word "alene" ('alone')

			represented an experience that seemed more straightforward and easily understood by the age group.
12.	ENG: Have you been happy with the way you are?<br>DA: Har du været glad for hvem du er?	DA: Har du været glad for at være dig?	The change in wording reflects a more accessible phrasing for younger children. In Danish "hvem du er" (who you are) is a more abstract concept that requires a certain level of self-awareness and understanding of identity. Meanwhile "at være dig" (to be you) simplifies the phrasing and focuses on the experience of being oneself. This is more relatable and concrete for young children, who may have a clearer sense of their everyday experiences in terms of connecting their feelings of happiness or satisfaction to their current state of being, rather than to articulate or even grasp what it means to evaluate their identity.
13.	ENG: Have you had enough time for yourself?<br>DA: Har du haft tid nok til dig selv?		
14.	ENG: Have you been able to do the things that you want to do in your free time?<br>DA: Har du kunnet gøre de ting, som du gerne ville i din fritid?		
15.	ENG: Have your parent(s) had enough time for you?<br>DA: Har dine forældre haft tid nok til dig?		
16.	ENG: Have your parent(s) treated you fairly?	Item excluded.	

	DA: Har dine forældre behandlet dig retfærdigt?		
17.	ENG: Have you been able to talk to your parent(s) when you wanted to?<br>DA: Har du kunnet tale med dine forældre, når du gerne ville?		
18.	ENG: Have you had enough money to do the same things as your friends?<br>DA: Har du haft penge nok til at gøre de samme ting som dine venner?	Item excluded.	
19.	ENG: Have you had enough money for your expenses?<br>DA: Har du haft penge nok til dine udgifter?	Item excluded.	
20.	ENG: Have you spent time with your friends?<br>DA: Har du været sammen med dine venner?		
21.	ENG: Have you had fun with your friends?<br>DA: Har du haft det sjovt med dine venner?		
22.	ENG: Have you and your friends helped each other?<br>DA: Har du og dine venner hjulpet hinanden?		
23.	ENG: Have you been able to rely on your friends?<br>DA: Har du kunnet regne med dine venner?	DA: Har du kunnet regne med/stole på dine venner?	The decision to add the phrase “stole på” (another Danish word for “rely on”) is based on that many of the children did not use or understand the phrase “regne med”. By combining both phrases, we enable more children understand the question.
24.	ENG: Have you been happy at school?		

	DA: Har du været glad i skolen?		
25.	ENG: Have you got on well at school?<br>DA: Har du klaret dig godt i skolen?	DA: Har du haft det godt i skolen?	The Danish phrase ”Har du klaret dig godt i skolen?” (Have you done well in school?) focuses on performance and achievements, implying an evaluation of a child's academic success and how well they have managed their schoolwork.<br>The rephrased item ”Har du haft det godt i skolen?” (directly translated to “Have you felt good/well at school?”) shifts the focus from academic performance to the emotional and social experience of getting on well in school. We believe that the adjustment is more age-appropriate and in line with the meaning of the original English version of the item for young children.
26.	ENG: Have you been able to pay attention?<br>DA: Har du været i stand til at være opmærksom?	DA: Har du kunnet koncentrere dig i skolen?	“At være opmærksom” directly translates to pay attention; however, in a Danish context, this phrase is not commonly interpreted in the same way as it is in English. In Danish, the term generally refers to a state of awareness or alertness, which can be broad and may simply imply noticing what is happening around them, particularly for young children.<br>When considering the terminology used to assess young children’s engagement in a school setting, we have therefore changed this phrase to “koncentrere sig” (to concentrate). This signifies a deeper level of focus and engagement with a specific task or activity. For young children concentrating often means sustaining attention on

			particular activities, such as listening to a teacher or working on a specific task.
27.	ENG: Have you got along well with your teachers?<br>DA: Har du været på god fod med dine lærere?	DA: Har du haft det godt sammen med dine lærere?	In Danish, the phrase “på god fod” (literally translated as ‘on good feet’) is an old idiomatic expression that means to be on good terms or to have a good relationship with someone – in this case your teachers. It is not commonly used among children and the children did not understand it.<br>While adults may understand and frequently use this idiom, children, especially younger children, might not be familiar with idiomatic expressions and knowing that children often struggle with abstracts concepts the use of such an idiom do not seem age-appropriate. Therefore, we changed the item to a more direct translation of the original English version of the item ”Har du haft det godt sammen med dine lærere?” (‘Have you got along well with your teachers?’).

Appendix 5. Information on item response distributions

Table 1a. Item response distributions for the KIDSCREEN-VIDEO

**Item**	**Response categories**				
	Never	*Seldom*	*Often*	*Very often*	*Always*
**1**	15	52	199	217	251
**2**	34	76	211	270	197
**3**	74	126	231	180	177
**4**	29	91	158	209	301
**5**	20	61	249	263	195
**6**	15	40	118	176	439
**7**	10	58	212	327	181
**8**	9	41	156	336	246
**9**	26	60	193	415	94
**10**	32	46	100	332	278
**11**	17	55	115	369	23
**12**	24	36	80	174	474
**13**	35	35	214	246	195
**14**	31	116	218	244	179
**15**	18	92	189	229	260
**16**	14	52	156	230	336
**17**	16	48	157	252	315
**18**	9	37	130	247	365
**19**	19	44	152	246	327
**20**	22	70	187	274	235
**21**	28	55	153	223	329
**22**	21	43	153	232	339
**23**	30	93	243	259	163
**24**	17	45	125	229	372

Table 1b. Item response distributions for the global and physical self-worth subscales

Item	Response categories					
	Completely disagree	Mostly disagree	Slightly disagree	Slightly agree	Mostly agree	Completely agree	I do not understand
GSW1	10	6	22	95	127	380	148
GSW2	18	6	46	110	116	433	59
PSW1	11	8	33	121	140	406	69
PSW2	11	6	18	129	144	419	61
